# Isometric or Isotonic Exercises in Alleviating Chronic Neck and Shoulder Pain and Enhancing Quality of Life Among Computer Users with Upper Crossed Syndrome: A Randomized Controlled Trial

**DOI:** 10.5812/aapm-160771

**Published:** 2025-05-13

**Authors:** Arash Khaledi, Hooman Minoonejad

**Affiliations:** 1 Department of Physical Education and Sport Sciences, Mashhad Branch, Islamic Azad University, Mashhad, Iran; 2 Department of Sports Injury and Biomechanics, Faculty of Sport Sciences and Health, University of Tehran, Tehran, Iran

**Keywords:** Upper Cross Syndrome, Exercise Therapy, Neck Pain, Shoulder Pain, Quality of Life

## Abstract

**Background:**

Millions of computer users experience chronic neck and shoulder pain (CNSP) and reduced health-related quality of life (HRQoL) due to upper cross syndrome (UCS). While strengthening exercises for the posterior trunk alleviate symptoms, it remains unclear whether isometric or isotonic exercises are more effective.

**Objectives:**

This study aimed to compare the effects of isometric and isotonic exercises on CNSP and HRQoL in individuals with UCS, and to evaluate these outcomes against a non-intervention group.

**Methods:**

In this randomized clinical trial (RCT), 43 UCS patients with CNSP were divided into three groups: Isometric exercises (n = 15), isotonic exercises (n = 14), and a control group (n = 14). Over 8 weeks, exercise groups completed 3 sessions per week (40 - 60 minutes each). Pain was assessed using the Visual Analog Scale (VAS) and HRQoL was assessed using the 36-item short form health survey (SF-36) questionnaire, both pre- and post-intervention.

**Results:**

Both isometric and isotonic exercises significantly reduced CNSP and improved HRQoL compared to the control group. Isometric exercises yielded a 70.4% pain reduction (P < 0.001) and a 14.9% HRQoL improvement (P = 0.002), while isotonic training showed a 47.6% pain reduction (P = 0.001) and a 17.7% HRQoL improvement (P < 0.001). Between-group differences were not statistically significant (pain: P = 0.853; HRQoL: P = 0.999). Although isometric exercises slightly favored pain reduction and isotonic exercises showed marginal HRQoL gains, these differences should not be overstated.

**Conclusions:**

Both isometric and isotonic exercises improved CNSP and HRQoL in UCS patients, with no significant difference between them. Slight trends favoring each should be interpreted cautiously. Longer-term studies are warranted.

## 1. Background

Technological advancements and increased post-COVID-19 screen time have fueled sedentary lifestyles, significantly contributing to the rising prevalence of upper crossed syndrome (UCS). Upper crossed syndrome is a musculoskeletal condition marked by forward head posture (cervical angle ≥ 45°), rounded shoulders (acromial angle ≥ 52°), elevated and protracted scapulae, and increased thoracic kyphosis (Cobb angle ≥ 42°), resulting from muscle imbalances (1, 2): Hypertonic pectorals, levator scapulae, and upper trapezius, alongside weak middle/lower trapezius and deep cervical flexors (3).

Epidemiological data further highlight the impact of UCS, particularly among sedentary office workers. Nejati et al. reported high incidences of forward head posture (FHP) (61.3%), rounded shoulders (48.7%), and hyperkyphosis (78.3%) in this population, associated with symptoms such as myofascial pain, paresthesia, restricted range of motion (ROM), and reduced health-related quality of life (HRQoL) (4). Similarly, prolonged computer use is strongly correlated with neck pain (affecting up to 67% of adults) (5), and shoulder pain (reported by 50.5% of users) (2).

To address UCS and its associated chronic neck and shoulder pain (CNSP), various therapeutic options are available, including physiotherapy, pharmacological treatments, behavioral interventions, targeted exercises, and surgery in severe cases (6, 7). Notably, therapeutic exercise is a prominent approach due to its cost-effectiveness, non-invasiveness, and capacity to correct underlying muscular imbalances (2, 6), demonstrating effectiveness in reducing nociceptive pain and improving HRQoL (2, 8).

Although various exercise approaches—such as stretching and strengthening—have proven beneficial (2, 6, 8), a 2024 review found no single best method (6). Strengthening may be superior to stretching for spinal deformities and pain (9, 10), but the optimal type (isometric vs. isotonic) is unknown. These simpler exercises are safer for those with poor physical conditioning (6, 9, 10).

Isometric exercises are static muscle contractions without joint movement, primarily engaging slow-twitch (type I) fibers in postural muscles (e.g., deep cervical flexors, lower trapezius). These muscles stabilize the body, but chronic shortening can lead to dysfunctional movement and pain. Conversely, isotonic exercises involve dynamic contractions with joint movement, activating fast-twitch (type II) fibers in phasic muscles (e.g., middle trapezius, rhomboids), crucial for force but prone to fatigue and weakness from disuse (11).

Despite growing interest and theoretical support (2, 9, 10), a crucial clinical question remains: Which type of strengthening exercise—isometric or isotonic—is more effective in alleviating CNSP and enhancing HRQoL in individuals with UCS? Identifying the optimal exercise type for this increasingly prevalent condition in sedentary populations could significantly improve rehabilitation and reduce musculoskeletal burden. While research exists, direct comparisons between isometric and isotonic strengthening for UCS are limited (6), highlighting the need for further investigation (2, 9, 10). Notably, no prior studies have directly compared these modalities in this specific context.

## 2. Objectives

(1) Assess the efficacy of isometric exercises compared to no intervention; (2) evaluate the efficacy of isotonic exercises compared to no intervention; (3) directly compare the effects of isometric and isotonic exercises on CNSP and HRQoL in individuals with UCS.

## 3. Methods

### 3.1. Study Design

This randomized clinical trial (RCT) was conducted at the University of Tehran's Faculty of Sports Sciences and Health Laboratory from June 2024 to March 2025, with ethical approval (IR.UT.SPORT.REC.1403.048) and in accordance with the Declaration of Helsinki. All participants provided written informed consent, and the trial was registered (IRCT20180727040609N3).

### 3.2. Participants

**Figure 1.**
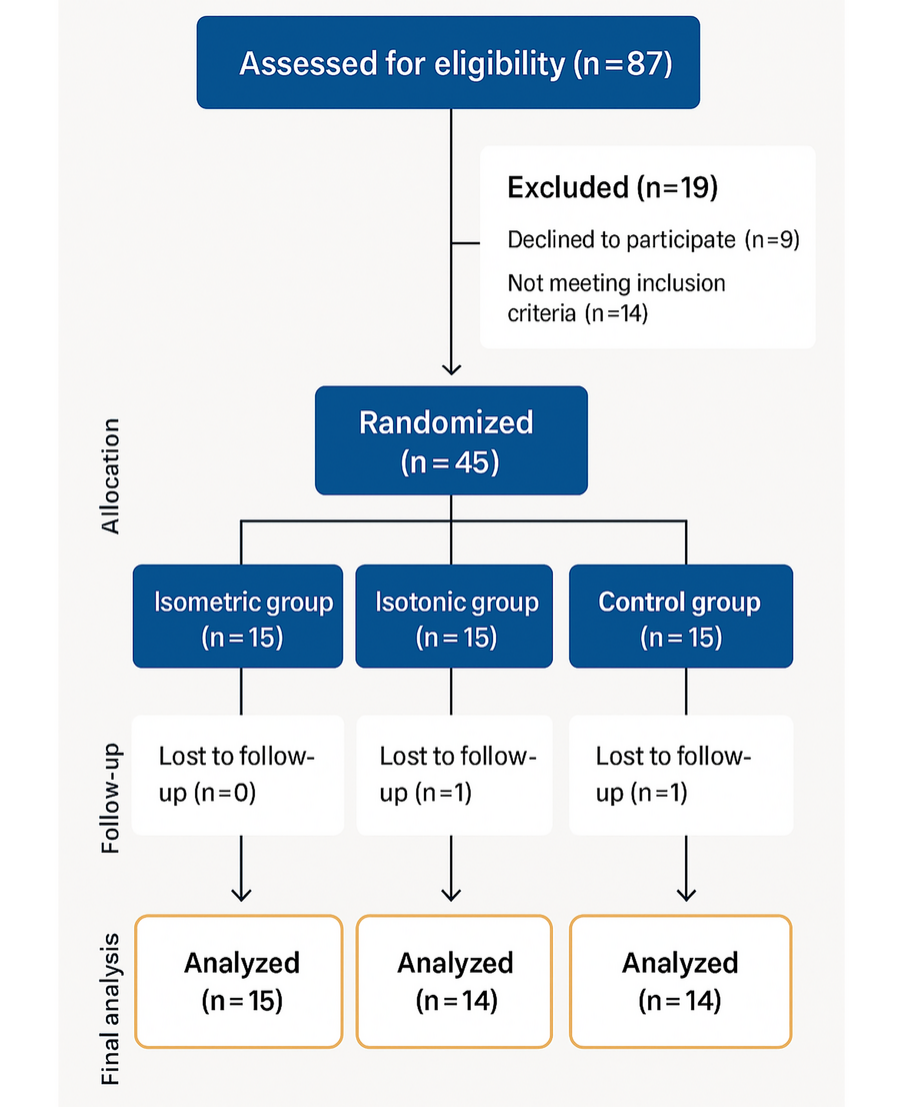
Flow diagram of the patients

Forty-three computer users with UCS and chronic neck/shoulder pain (≥ 3 hours/day computer use for ≥ 3 years) (12) completed the 8-week intervention. Participants were randomized into two intervention groups (isometric, n = 15; isotonic, n = 14) and one control group (n = 14) using Research Randomizer. Power analysis (G*Power 3.1, 80% power, α = 0.05, effect size = 0.88) (13) recommended 30 participants; 45 were recruited to account for attrition (2 dropped out) (Figure 1). 

### 3.3. Inclusion Criteria

Participants [aged 20 - 60, body mass index (BMI) ≤ 30] had postural deviations characterized by forward head posture (≥ 45°), rounded shoulders (≥ 52°), and rounded back (≥ 42°). They reported computer use of more than 3 hours per day for at least 2 years and experienced chronic neck/shoulder pain with a Visual Analog Scale (VAS) score of ≥ 3 for a duration of at least 3 months (2).

### 3.4. Exclusion Criteria

Exclusions included recent treatments for CNSP within the past 3 months (7), non-mechanical causes of CNSP, spinal pathologies (e.g., fractures, surgery, inflammation), systemic diseases (e.g., fibromyalgia, osteoporosis), pregnancy, continuous use of pain medication, or non-compliance with the study protocol (attendance of less than 90%) (2, 13).

### 3.5. Outcome Measures

#### 3.5.1. Assessment of Pain Intensity

Pain intensity was assessed using a 100-mm VAS. Participants indicated their current pain level by marking a point on a line that ranges from 0 (representing no pain) to 10 (representing the worst imaginable pain). This measurement tool has demonstrated strong inter-rater reliability (ICC = 1.00) and test-retest reliability (ICC = 0.99) in previous studies (14).

#### 3.5.2. Assessment of the Level of Health-Related Quality of Life

Health-related quality of life was assessed using the validated Persian version of the 36-item short form health survey (SF-36). This tool evaluates self-perceived health across eight domains: (1) Physical functioning; (2) role limitations due to physical health; (3) role limitations due to emotional problems; (4) energy/fatigue; (5) emotional well-being; (6) social functioning; (7) bodily pain; and (8) general health. Participants received clear instructions and sufficient time to complete the survey. Scores range from 0 to 100, with higher scores reflecting better health status. The SF-36 has demonstrated good validity (70 - 85%) and reliability (Cronbach’s α = 0.65 - 0.90) in Iranian populations (15).

#### 3.5.3. Interventions

Participants were divided into three groups: A control group and two intervention groups (isometric and isotonic exercise). All participants received standardized educational materials on postural hygiene via a booklet to control for attention/time effects and ensure ethical parity. Additionally, the intervention groups received a second booklet outlining their specific exercise protocols. The control group was placed on an 8-week waitlist and monitored for changes, in line with ethical guidelines (5, 11).

The two intervention groups completed an 8-week exercise therapy program, three times a week. Sessions included a 5 - 7 minute warm-up, 40 - 50 minute main exercise, and 3 - 5 minute cool-down, totaling 40 - 60 minutes, following FITT principles (Appendix 1 Supplementary File) (5). The first session focused on teaching correct movements using demonstrations, images, and videos. The second session was supervised by a specialist to ensure proper execution. Participants then continued independently at home, with adherence monitored via bi-weekly online check-ins and phone follow-ups. They reported exercise frequency, duration, challenges, and compliance. Non-adherent participants were excluded, ensuring a per-protocol analysis.

**Figure 2.**
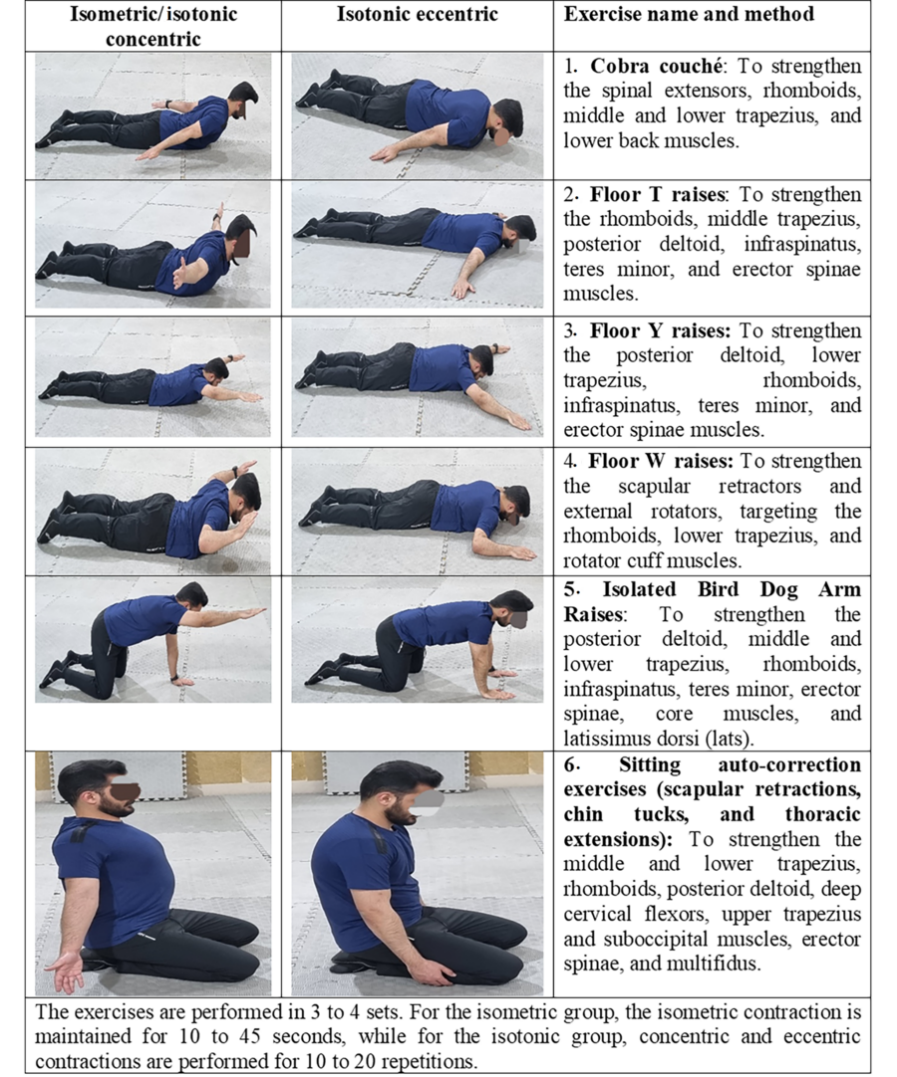
Isometric and isotonic exercise program

Both groups performed six exercises targeting shoulder, neck, and thoracic spine extensor muscles: (1) Cobra Couché; (2) floor T raises; (3) floor Y raises; (4) floor W raises; (5) isolated bird dog arm raises; and (6) sitting auto-correction exercises (scapular retractions, chin tucks, thoracic extensions) (16). The isometric group held static positions, while the isotonic group performed dynamic movements (11) (Figure 2). 

### 3.6. Statistical Analyses

To describe the data in this study, descriptive statistical indicators such as percentages, mean, and standard deviation were used. In the inferential statistics section, analysis of covariance (ANCOVA) was applied to test the hypotheses. Finally, the Bonferroni post hoc test was used to compare groups at a significance level of 0.05.

## 4. Results

### 4.1. Participant Characteristics

Table 1 summarizes patient characteristics across the three groups. The ANOVA revealed no statistically significant differences between groups for age (P = 0.544), weight (P = 0.059), height (P = 0.775), or BMI (P = 0.224).

**Table 1.** Comparison of Patients’ Characteristics Between Groups

Variables	Isometric Group (n = 15)	Isotonic Group (n = 14)	Control Group (n = 14)	P-Value
**Age (y)**	39.13 ± 7.15	36.86 ± 9.71	35.86 ± 7.43	0.544 ^NS^
**Weight (kg)**	74.80 ± 9.75	77.29 ± 9.37	76.29 ± 9.08	0.059 ^NS^
**Height (cm)**	162.07 ± 8.51	171.21 ± 12.08	168.86 ± 10.51	0.775 ^NS^
**BMI (kg/m** ^**2**^ **)**	28.67 ± 4.73	26.41 ± 2.26	26.89 ± 3.45	0.224 ^NS^

Abbreviations: NS, non-significant; BMI, body mass index.

### 4.2. Pain Scores

Numerically, the isometric group demonstrated the lowest pain levels (3.38 ± 0.33), followed by the isotonic group (3.90 ± 0.35), while the control group reported the highest pain scores (5.76 ± 0.35). Although this pattern suggests a potential trend favoring isometric exercises for pain reduction, statistical analysis indicated no significant difference between the two intervention groups (P = 0.853). However, ANCOVA revealed a significant overall difference in pain scores among the groups (F (2, 39) = 13.37, P < 0.001, η^2^ = 0.41).

Post-hoc comparisons showed that both the isometric and isotonic groups had significantly lower pain than the control group (isometric vs. control: P < 0.001, 70.4% reduction, 95% CI: -3.59 to -1.17; isotonic vs. control: P = 0.001, 47.6% reduction, 95% CI: -3.08 to -0.64). In contrast, no significant difference was observed between the isometric and isotonic groups (P = 0.853, 15.4% difference, 95% CI: -1.73 to 0.68) (Table 2). 

**Table 2.** Results of Bonferroni Post-Hoc Test ^a^

Variables and Intervention Time	Isometric (n = 15)	Isotonic (n = 14)	Control (n = 14)	P-Value
**Pain (score)**				< 0.001
Pre	5.60 ± 1.55	5.93 ± 1.69	5.93 ± 1.64	
Post	3.38 ± 0.033 ^b^	3.90 ± 0.35 ^b^	5.76 ± 0.35	
**SF-36 (score)**				< 0.001
Pre	43.73 ± 4.82	43.71 ± 5.95	42.57 ± 3.99	
Post	53.11 ± 1.42 ^b^	51.39 ± 1.47 ^b^	43.71 ± 1.47	

Abbreviation: SF-36, 36-item short form health survey.

^a^ Values are expressed as mean ± SD.

^b^ P ≤ 0.001 significant difference with control group.

### 4.3. Health-Related Quality of Life

**Figure 3.**
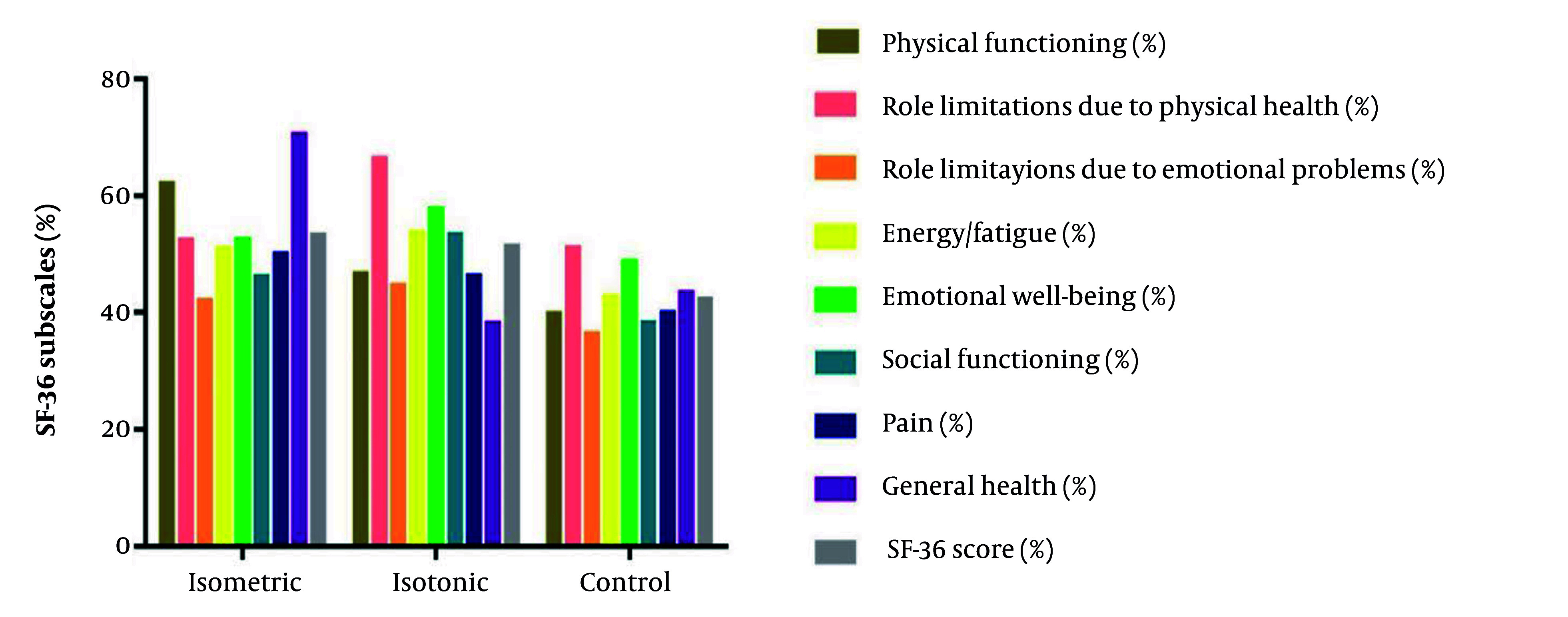
The subscales for 36-item short form health survey (SF-36) health-related quality of life in patients with upper cross syndrome after the intervention

Figure 3 displays the SF-36 HRQoL outcomes across all eight subscales. Numerically, the isotonic exercise group demonstrated the greatest improvements, showing benefits across all measured domains, including physical functioning, pain reduction, and mental health. The isometric group exhibited moderate yet meaningful improvements, particularly in the general health and physical limitation subscales, though to a lesser degree than the isotonic group. In contrast, the control group showed minimal changes across all HRQoL dimensions.

The ANCOVA revealed statistically significant between-group differences in overall HRQoL scores (F (2, 39) = 11.79, P < 0.001, η^2^ = 0.38). Post-hoc analysis confirmed that both exercise interventions significantly improved HRQoL compared to the control group (isotonic: P < 0.001, 17.7% improvement, 95% CI: 2.48 to 12.88; isometric: P = 0.002, 14.9% improvement, 95% CI: 4.29 to 14.51). However, direct comparison between the two exercise modalities showed no statistically significant difference (P = 0.999, 3.2% difference, 95% CI: -3.37 to 6.81).

## 5. Discussion

This study compared isometric and isotonic exercises for CNSP and HRQoL in computer users with UCS. Over eight weeks, isometric exercises reduced pain more effectively, while isotonic exercises slightly improved HRQoL. Both exercise interventions outperformed the control group, with no significant differences between them. The findings highlight the benefits of tailored exercise for UCS.

Both exercise groups showed significant pain reduction compared to controls—70.4% for isometric (P < 0.001) and 47.6% for isotonic (P = 0.001). Although the isometric group had a greater effect (15.4% difference), the between-group difference was not significant (P = 0.853). These findings align with prior studies on neck (2, 17-19) and shoulder pain (2, 20, 21). The non-significant trend favoring isometric exercises aligns with findings from some studies (11, 19), suggesting that pain relief in UCS may involve complex biomechanical and neurophysiological mechanisms.

Prolonged computer use demands sustained activation of postural stabilizers (e.g., trapezius, levator scapulae, rhomboids), making isometric exercises highly relevant. These exercises enhance strength and local endurance without joint motion, benefiting those with chronic pain or hypermobility by reducing nociceptive input and microtrauma risk (2, 19, 20). Unlike concentric/eccentric loading, isometric exercises enable targeted recruitment without aggravating tendinopathy, bursitis, or myofascial pain (11, 20, 21). The lack of cyclic loading also lowers overuse injury risks (e.g., tendinosis, stress fractures), crucial for pain-sensitive individuals with potential central/peripheral sensitization (20, 22).

Comparative studies offer additional insights. Kinsella et al. found comparable effects for isotonic and isometric exercises in subacromial pain syndrome and rotator cuff tendinopathy, likely due to isotonic exercises' functional nature (20). However, their population involved repetitive motion injuries, unlike UCS, which arises from sustained poor posture. In contrast, Fatima et al. reported superior efficacy of isometrics for subacromial impingement, linking it to reduced rotator cuff stress, aligning with our findings (22).

Regarding HRQoL, both interventions significantly improved SF-36 scores versus controls. While the isotonic group demonstrated a slightly higher percentage improvement (17.7%, P < 0.001) than the isometric group (14.9%, P = 0.002), this comparison is based on change scores rather than absolute post-intervention values, and the difference between the two intervention groups was not statistically significant (P = 0.999). These findings align with previous studies on strengthening exercises and HRQoL (6, 23, 24). However, the unblinded design may have introduced bias in subjective measures, as the isotonic group's preference for dynamic movements could have influenced self-reports.

Lederman maintains that dynamic-active exercises (e.g., isotonic) more effectively activate sensorimotor systems and enhance proprioceptive feedback, thereby improving functional performance (25). In contrast, pain reduction appears to exert a more pronounced effect on HRQoL through its facilitation of daily activities (26). This distinction clarifies why both exercise modalities yielded similar HRQoL benefits, notwithstanding their differences in movement complexity and neuromuscular requirements.

The 8-week study may have been too short to identify long-term effects, and a longer duration (e.g., 12+ weeks) could provide clearer insights. However, since pain and quality-of-life improvements were similar between groups, neither exercise type showed clear superiority. Larger, longer-term RCTs are needed for more definitive conclusions.

The study has several limitations:

(1) Uncontrolled daily environments may have affected outcomes (2).

(2) The short duration limits long-term efficacy assessment.

(3) Lack of blinding risks performance/detection bias.

(4) Physiological mechanisms (e.g., muscle activation) were not examined.

(5) The reliance on subjective measures without objective assessments (e.g., posture analysis, muscle strength testing) limits the robustness of the conclusions.

Addressing these limitations in future studies would improve the evidence and practical application.

### 5.1. Conclusions

Both isometric and isotonic exercises effectively reduced CNSP and improved HRQoL in computer users with UCS compared to the control group. Although isometric exercise demonstrated a trend toward greater pain reduction and isotonic exercise showed a marginal HRQoL improvement, these differences were not statistically significant and should be interpreted cautiously. Future studies with larger sample sizes and longer durations are needed to determine whether one approach is superior. Clinically, however, both exercise types appear beneficial for alleviating UCS symptoms.

aapm-15-3-160771.pdf

## Data Availability

The dataset presented in the study is available on request from the corresponding author during submission or after its publication. The data are not publicly available due to privacy concerns and ethical restrictions related to participant confidentiality.

## Clinical Trial Registration Code:

IRCT20180727040609N3.
